# Selective Expression of a SNARE-Cleaving Protease in Peripheral Sensory Neurons Attenuates Pain-Related Gene Transcription and Neuropeptide Release

**DOI:** 10.3390/ijms22168826

**Published:** 2021-08-17

**Authors:** Wanzhi Wang, Miaomiao Kong, Yu Dou, Shanghai Xue, Yang Liu, Yinghao Zhang, Weiwei Chen, Yanqing Li, Xiaolong Dai, Jianghui Meng, Jiafu Wang

**Affiliations:** 1 School of Life Sciences, Henan University, Kaifeng 475001, China; 104753180826@vip.henu.edu.cn (W.W.); 104753190784@henu.edu.cn (M.K.); 104753180809@vip.henu.edu.cn (Y.D.); 104753190789@henu.edu.cn (S.X.); 104753180810@vip.henu.edu.cn (Y.L.); 104752190124@henu.edu.cn (Y.Z.); 104752180086@vip.henu.edu.cn (W.C.); 104752190132@henu.edu.cn (Y.L.); 104753201015@henu.edu.cn (X.D.); 2 School of Biotechnology, Faculty of Science and Health, Dublin City University, Glasnevin, Dublin 9, Ireland

**Keywords:** SNARE, cytokine, dorsal root ganglia, neuropeptide, Pirt, neuronal promoter, gene therapy, neurotoxin, chronic pain

## Abstract

Chronic pain is a leading health and socioeconomic problem and an unmet need exists for long-lasting analgesics. SNAREs (soluble N-ethylmaleimide-sensitive factor attachment protein receptors) are required for neuropeptide release and noxious signal transducer surface trafficking, thus, selective expression of the SNARE-cleaving light-chain protease of botulinum neurotoxin A (LCA) in peripheral sensory neurons could alleviate chronic pain. However, a safety concern to this approach is the lack of a sensory neuronal promoter to prevent the expression of LCA in the central nervous system. Towards this, we exploit the unique characteristics of Pirt (phosphoinositide-interacting regulator of TRP), which is expressed in peripheral nociceptive neurons. For the first time, we identified a Pirt promoter element and cloned it into a lentiviral vector driving transgene expression selectively in peripheral sensory neurons. Pirt promoter driven-LCA expression yielded rapid and concentration-dependent cleavage of SNAP-25 in cultured sensory neurons. Moreover, the transcripts of pain-related genes (*TAC1, tachykinin precursor 1;* *CALCB, calcitonin gene-related peptide 2; HTR3A, 5-hydroxytryptamine receptor 3A;* *NPY2R, neuropeptide Y receptor Y2;* *GPR52, G protein-coupled receptor 52;* *SCN9A, sodium voltage-gated channel alpha subunit 9;* *TRPV1* and *TRPA1, transient receptor potential cation channel subfamily V member 1* and *subfamily A member 1)* in pro-inflammatory cytokines stimulated sensory neurons were downregulated by viral mediated expression of LCA. Furthermore, viral expression of LCA yielded long-lasting inhibition of pain mediator release. Thus, we show that the engineered Pirt-LCA virus may provide a novel means for long lasting pain relief.

## 1. Introduction

It is estimated that ~1.5 billion people worldwide suffer from chronic pain [1], which causes a major healthcare challenge and huge societal burden. More than 70% of cancer patients are suffering cancer-related pain. Despite the fact that chronic pain significantly affects patients’ quality of life, the number of efficacious options is limited. Conventional anti-inflammatory drugs (non-steroidal and steroidal) and painkillers (opiates) are short-lived, inadequate, merely palliative, and are usually associated with intolerable side-effects (addiction and sometimes life-threatening) [2,3]. Hence, there is a major unmet need for long-lasting and non-addictive therapeutics.

Chronic pain can be neuropathic, which includes pain caused by direct injury to peripheral or central nerves, or inflammatory (e.g., rheumatoid arthritis pain). The advent of the biotherapeutics has now tremendously changed the approach to treatment. Administering recombinant IL-1 receptor antagonist or monoclonal antibodies against TNF-α or IL-6 or their receptors has proved beneficial in clinical therapy of patients with rheumatoid arthritis [4,5,6]. Injection of tanezumab, an anti-NGF (nerve growth factor) monoclonal antibody, could antagonize the proalgesic action of NGF to relieve osteoarthritic pain [7,8,9]. However, some participants with osteoarthritis experienced joint disintegration [8,9]. The small-molecule drug discovery ecosystem has changed radically over the last decade. Blockers of certain channels responsible for pain transduction and amplification (e.g., the transient receptor potential cation channels (TRP), voltage-gated sodium channels 1.7 (Na_v_1.7) (encoded by *SCN9A* gene) have also been used in clinical trials for the management of pain [10,11]. The development of opioid receptor agonists (such as Oliceridine, BU10038) with reduced side effects (e.g., constipation and cessation of breathing, addiction and dependence liabilities) as pain killers has advanced [12,13]. Although these therapeutics have yielded encouraging results, their short-duration of action, numerous adverse reactions, and diminishing efficacy over time emphasize the urgent need for improved versions.

A new strategy has emerged using botulinum neurotoxins (BoNTs) as a local treatment for chronic pain. BoNTs are the most powerful inhibitor of the exocytosis of neurotransmitters. Intra-articular injection of BoNT/A complex can reduce joint pain severity with a significant improvement in function [14,15,16]. Moreover, BoNT/A complex (BOTOX) can also alleviate headache pain symptoms for a portion of migraine patients [16,17,18]. Currently, botulinum toxin injections have been US FDA approved for chronic migraines. There are seven serotypes of BoNTs (BoNT/A to BoNT/G) existing in nature [16,19,20,21,22,23,24]. In 2017, a new serotype termed as BoNT/X was discovered using bioinfomatics analysis approach [25]. BoNTs consist of a ~50 kDa light chain protease (LC) linked to a ~100 kDa heavy chain (HC) via a disulfide bond. HC is responsible for binding neurons and translocation of the attached LC to the cytosol, wherein LC protease truncates and inactivates soluble N-ethylmaleimide-sensitive factor attachment protein receptors (SNAREs). BoNT/A inactivates synaptosomal associated protein 25-kDa (SNAP-25) by removing nine amino acids from its C-terminal. Cleavage of SNAP-25 results in the blockade of the vesicle fusion and neurotransmitter release [20,21,22,26,27,28,29]. BoNT/A can enter sensory neurons and block pain-related neuropeptide release (such as substance P and calcitonin gene-related peptide (CGRP)) [30,31]. Moreover, BoNT/A can lower the surface delivery of important pain-perception receptors and transducing channels (such as TRPV1, TRPA1 and Na_v_1.7 etc) and the resultant hyper-sensitization in inflammation [32,33]. As an analgesic agent, BoNT/A has the advantage of being non-additive and disadvantage of producing unwanted side effects (e.g., muscle paralysis) and risk of the reported central effects [34,35,36].

We are in urgent need of new therapies for long-term pain management and gene transfer of BoNT LC protease into peripheral sensory neurons provides a novel means to alleviate chronic pain. Current viral vectors used for gene therapy usually have a broad tropism; therefore, they can deliver the encoded therapeutic gene to a wide spectrum of cells resulting in unwanted off-target effects. To avoid the unwanted transfer of the LC of BoNT/A (LCA) into motor neurons to prevent muscle paralysis side effect, it is ideally to use a sensory neuronal promoter to drive the transgene expression. However, specific sensory neuronal promoter has not been identified. A previous study reported that a membrane protein named Pirt (phosphoinositide interacting regulator of TRP), known to play a vital role in sensing pain through modulation of the TRPV1, is expressed specifically in the peripheral nervous system, predominantly in dorsal root ganglion (DRG) and trigeminal ganglion (TG) nociceptive neurons [37]. Strikingly, Pirt is not expressed in the central nervous system [37]. Thus, Pirt gene promoter fulfils the criteria for selective expression of analgesic transgene in the sensory neurons for management peripheral derived pain. Herein, we firstly cloned the predicated promoter sequence into a lentiviral transfer vector. Subsequently, LCA gene and GFP reporter gene were separately arranged into the downstream of Pirt promoter to produce lentiPirtLCA and lentiPirtGFP virus, respectively. Pirt promoter could drive transgene expression (GFP and LCA) selectively in the peripheral sensory neurons. Viral mediated LCA expression in the cultured sensory neurons leads to effective cleavage of SNAP-25. In contrast, LCA proteolytic activity was hardly detected in the infected spinal cord neurons, reassuring Pirt specificity in peripheral sensory neurons. Of note, viral-mediated LCA expression lowered the mRNA levels of certain neuropeptide, receptor and transducer genes in proinflammatory cytokines stimulated sensory neurons. Most importantly, lentiPirtLCA-induced persistent cleavage of SNAP-25 and inhibition of neuropeptide release in the infected sensory neurons with potential to yield long-lasting relief of chronic pain in patients.

## 2. Results

### 2.1. Creation of a Novel Expression Cassette Using Pirt Promoter to Drive Therapeutic Gene Expression Selectively in Peripheral Sensory Neurons

**Figure 1:**
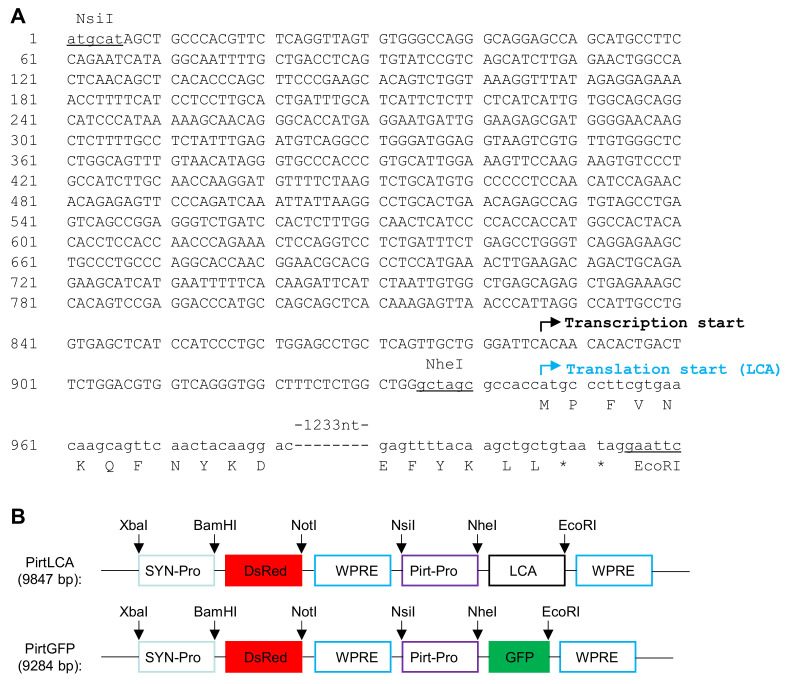
Illustration of the engineered lentiviral transfer vectors. (**A**) Nucleotide sequence of PirtLCA expression cassette. Pirt promoter nucleotides are depicted in capital letters. Underlined nucleotides are engineered restriction enzyme recognition sequences for cloning. The predicated transcription start site and LCA translation initiation site are indicated. (**B**) Restriction map of PirtLCA and PirtGFP transfer vectors. The critical restriction sites are indicated with arrows. DsRed and GFP are referred to the two reporter genes.

The number of clinical trials involving adeno-associated virus, lentiviral vector and other retrovirus for gene therapy continues to increase every year. In order to express therapeutic gene selectively in peripheral sensory neurons, there is an unmet need to identify a suitable sensory neuronal promoter. Pirt gene is preferentially expressed in peripheral nociceptive neurons, its promoter can be exploited to express extraneous therapeutic gene in the sensory neurons to selectively attenuate pain-mediator release and normalize the expression of pain-related receptors and transducers. As the promoter sequence of Pirt gene is not identified, we analyzed the human Pirt gene using the Ensembl tool and selected a 928-bp nucleotide sequence as the predicated promoter element, which lacks a TATA box. Figure 1A shows the nucleotide sequence of the predicated Pirt promoter and the transcription starting site is displayed. A previously reported lentiviral vector (Syn-DsRed-Syn-GFP), which can express two reporter genes (DsRed and GFP) under two separated synapsin 1 (Syn) gene promoters [38], was used to construct new vectors. The Pirt promoter was synthesized and inserted into the lentiviral vector between NsiI and NheI sites to replace the second synapsin 1 gene promoter, this yielded a control plasmid which encodes GFP gene under Pirt promoter, named as PirtGFP (Figure 1B). Subsequently, nucleotide sequence encoding LCA was inserted into the PirtGFP vector between NheI and EcoRI sites to replace the GFP gene. This resulted in a new lentiviral vector (PirtLCA) (Figure 1A,B). All of these vectors were subjected to DNA sequencing to verify the accuracy of the promoter and inserts. The respective lentiviruses, lentiPirtLCA and lentiPirtGFP, were successfully generated in 293T cells by co-transfection of the constructed vector and packaging (psPAX2) and envelope (pMD2.G) plasmids. After concentration of the harvested supernatants by ultracentrifugation, the titers obtained for lentiPirtLCA and lentiPirtGFP are 2 × 10^7^ and 5 × 10^7^ transducing units (TU)/mL, respectively.

### 2.2. Pirt Promoter Selectively Drove Exogenous Gene Expression in Peripheral Sensory Neurons

**Figure 2:**
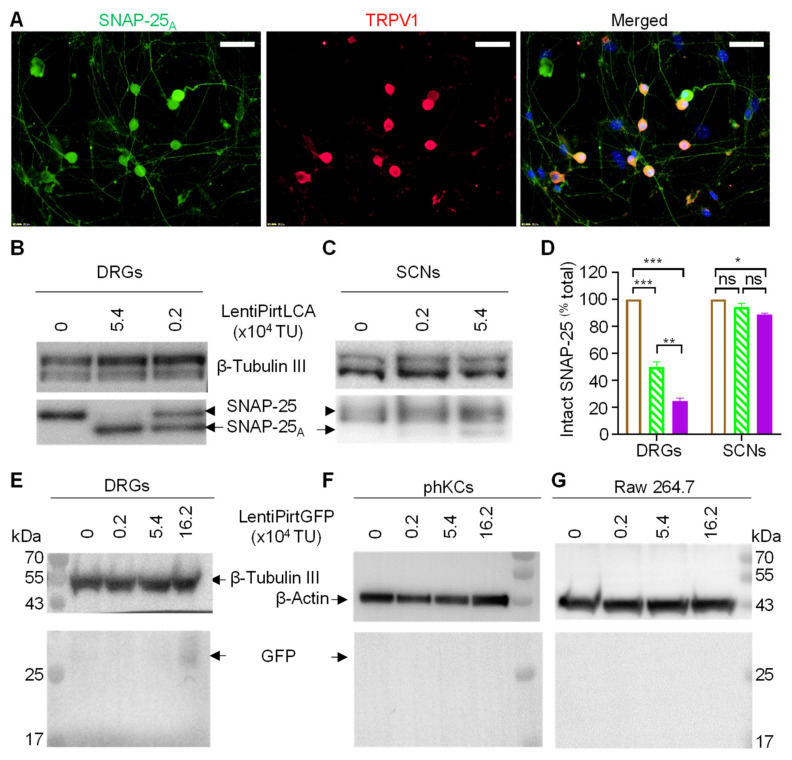
Pirt promoter drove exogenous gene expression selectively in sensory neurons. (**A**) Cultured mouse DRGs were incubated with 5.4 × 10^4^ TU/well for 5 days before performing double immunofluorescent staining with antibodies for the LCA cleaved SNAP-25_A_ (green) and TRPV1 (red). The nuclei were stained blue with 4′, 6-Diamidino-2-phenylindole dihydrochloride. Bars, 50 μm. Cultured mouse DRGs (**B**) and spinal cord neurons (SCNs) (**C**) were infected by lentiPirtLCA with the indicated doses for 5 days before probing the SNAP-25 cleavage by the expressed LCA. Intact SNAP-25 and LCA cleaved form were probed by Western blotting with an antibody recognizing both intact and cleaved forms. (**D**) Quantified data derived from densitometric scanning of 3 replicate gels. Empty bars, virus free; hatched bars, 0.2 × 10^4^ TU; filled bars, 5.4 × 10^4^ TU. Data presented are mean ± SEM, from three independent cultures. One-way ANOVA with Bonferroni’s multiple comparison test demonstrated significant differences between the groups in DRGs: virus free vs. 0.2 × 10^4^ TU *p* = 0.00003; virus free vs. 5.4 × 10^4^ TU *p* < 0.00001; 0.2 × 10^4^ TU vs. 5.4 × 10^4^ TU *p* = 0.00119. In SCNs: virus free vs. 5.4 × 10^4^ TU *p* = 0.01327. * *p* < 0.05; ** *p* < 0.01; *** *p* < 0.001, *p* > 0.05. Mouse DRGs (**E**), primary human keratinocytes (phKCs) (**F**) and Raw 264.7 cells (**G**) were infected with lentiPirtGFP for 5 days before probing the expression of GFP protein by Western blotting. β-tubulin III and β-actin proteins were probed as loading controls.

As BoNT LC is a potent protease, we used the LCA gene instead of widely used luciferase reporter gene to test the activity and neuronal tissue specificity of Pirt promoter. Cultured murine dorsal root ganglionic neurons (mDRGs) at 5 days in vitro in 24 well plates were infected by lentiPirtLCA at 5.4 × 10^4^ TU per well, equaling multiplicity of infection (MOI) of ~5.4. Cells were continually cultured for 5 days to allow the virus to express LCA before examining the cleavage of SNAP-25 in the infected cells by immunofluorescence staining with an antibody specific for the LCA cleaved form. Representative images indicated that all of the TRPV1 positive neurons were infected with virus and the cleaved SNAP-25 was detected in the cell bodies and fibers of TRPV1^+^ neurons (Figure 2A). To quantify the extent of SNAP-25 cleavage, mDRGs were infected by two different doses of lentiPirtLCA. The extent of cleavage was monitored by Western blotting using an antibody recognizing both the intact and cleaved SNAP-25. Expression of LCA was easily detected in the cells transduced with lentiPirtLCA at 0.2 × 10^4^ TU, as reflected by the efficient cleavage of SNAP-25 (Figure 2B,D). Higher amount of virus (5.4 × 10^4^ TU) cleaved majorities of the SNAP-25 (Figure 2B,D). In great contrast, lentiPirtLCA at 0.2 × 10^4^ TU dose did not induce any cleavage of SNAP-25 in cultured spinal cord neurons (SCNs) whereas higher dose of virus at 5.4 × 10^4^ TU only produced negligible cleavage of SNAP-25 even at day 5 after infection (Figure 2C,D).

As non-neuronal cells do not express SNAP-25, thus, we used lentiPirtGFP virus to infect two different types of cells, the cultured primary human keratinocytes (phKCs) and RAW264.7 macrophage cells to check the GFP reporter expression in these cells. Expression of GFP protein was probed by Western blotting using a GFP antibody. GFP protein was only detected in the lentiPirtGFP infected mDRGs (Figure 2E) but not in the tested phKCs (Figure 2F) and RAW264.7 cells (Figure 2G). Of note, promoter activity seems low as bright fluorescence was not detected under fluorescence microscopy. Nevertheless, our results confirm the preferential activity of Pirt gene promoter in peripheral sensory neurons but not in the other tested cells.

### 2.3. Pirt Promoter-Driven Expression of LCA Induced Rapid and Concentration Dependent SNAP-25 Cleavage in Peripheral Sensory Neurons

**Figure 3:**
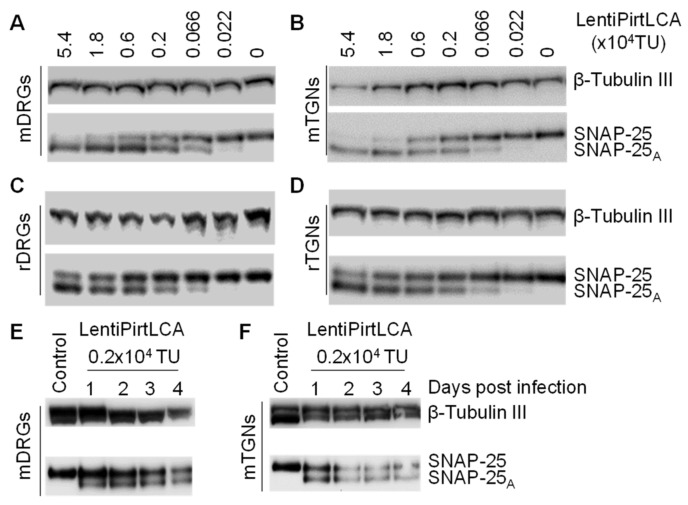
LentiPirtLCA yielded rapid and concentration dependent SNAP-25 cleavage in the infected sensory neurons. Cultured mDRGs (**A**), mTGNs (**B**), rat DRGs (rDRGs) (**C**) and rat TGNs (rTGNs) (**D**) were infected with lentiPirtLCA with the indicated doses for 5 days before probing the SNAP-25 cleavage. Western blots revealed lentiPirtLCA dose-dependently cleaved SNAP-25 in both DRGs and TGNs. mDRGs (**E**) and mTGNs (**F**) were incubated with lentiPirtLCA with the indicated dose for 24 h. Cells were either immediately harvested in LDS-sample buffer or continually cultured until the indicated time points before harvesting for probing the SNAP-25 cleavage. Control samples in (**E**,**F**) were virus-free samples at day 1.

Next, the activity of lentiPirtLCA was further assessed in vitro, using mDRGs. The latter were transduced by a series of diluted doses of lentiPirtLCA and the cleavage of SNAP-25 was probed. Representative blots demonstrated that the majorities of SNAP-25 were cleaved in mDRGs infected by virus at ≥1.8 × 10^4^ TU/well. Not surprisingly, the cleavage of SNAP-25 is infection dose dependent (Figure 3A). Similar experiments were repeated with cultured murine trigeminal ganglionic neurons (mTGNs). Again, lentiPirtLCA dose-dependently cleaved SNAP-25 in mTGNs (Figure 3B). As the Pirt gene is very conserved between human and rodents. It is our interest to investigate whether human Pirt gene promoter is also able to express the LCA gene in rat sensory neurons. Rat DRGs (rDRGs) and rat TGNs (rTGNs) were incubated with lentiPirtLCA before measuring the cleavage of SNAP-25 at day 5. Western blotting results indicated human Pirt promoter was able to drive the expression of LCA gene as revealed by the cleavage of SNAP-25 in rDRGs (Figure 3C) and rTGNs (Figure 3D).

Next, we checked the time course of appearance of SNAP-25 cleavage in mDRGs and mTGNs following infection by lentiPirtLCA. Cultured neurons were infected by virus at 0.2 × 10^4^ TU/well. The cleavage of SNAP-25 was readily detected within 24h in both mDRGs (Figure 3E) and mTGNs (Figure 3F). Prolonged incubation of virus did not yield more SNAP-25 cleavage, suggesting lentiviral-mediated expression of LCA can cleave the majority of SNAP-25 in the infected cells within 24 h. Thus, Pirt promoter-driven expression of LCA induced rapid and concentration dependent SNAP-25 cleavage in sensory neurons.

### 2.4. Viral-Mediated LCA Expression Downregulated Transcription Levels of Certain Neuropeptide, Receptor and Transducer Genes in Pro-Inflammatory Cytokines Stimulated mDRGs

**Figure 4:**
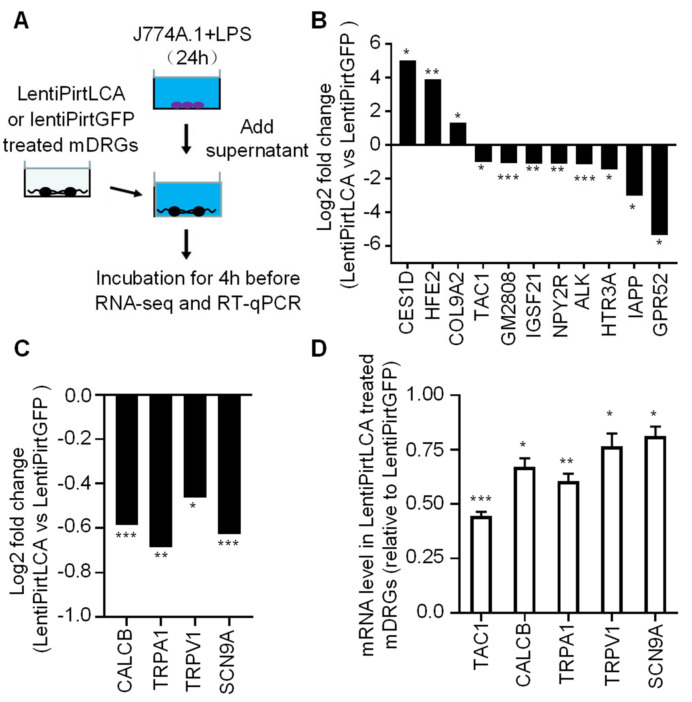
Viral-mediated LCA expression downregulated the mRNA levels of certain neuropeptide, receptor and transducer genes. (**A**) Schematic diagram of experimental setup. J774A.1 macrophage cells grown in 24 well plates were stimulated by 100 ng/mL lipopolysaccharide (LPS) for 24 h. The supernatant containing various cytokines was collected. mDRGs grown in 24 well plates were treated with lentiPirtLCA or lentiPirtGFP at 5.4 × 10^4^ TU/well for 5 days. Cells were then incubated with a mixture of the collected LPS-stimulated J774A.1 supernatant and DRG culture medium (500 µL each) for 4 h before being collected for RNA-seq (**B**,**C**) and RT-qPCR (**D**). RNA-seq results revealed that viral-mediated LCA expression significantly affected the transcriptional levels of 11 indicated genes (**B**) in comparison to viral mediated GFP expression, with less effective for *CALCB*, *TRPA1*, *TRPV1* and *SCN9A* genes (**C**). RNA-seq samples were from three independent cultures. FDR for *CES1D*, *HFE2*, *COL9A2*, *TAC1*, *GM2808*, *IGSF21*, *NPY2R*, *ALK*, *HTR3A*, *IAPP*, *GPR52*, *CALCB*, *TRPA1*, *TRPV1* and *SCN9A* are 0.0377, 0.0087, 0.0443, 0.0160, 3.8E-08, 0.0012, 0.0010, 8.54E-04, 0.0232, 0.0335, 0.0158, 1.20E-10, 0.0030, 0.01828746 and 6.78E-12, respectively. * 0.01 < FDR < 0.05, ** 0.001 < FDR < 0.01, *** FDR < 0.001. (**D**) RT-qPCR results revealed that the mRNA levels of *TAC1*, *CALCB*, *TRPA1*, *TRPV1* and *SCN9A* genes were downregulated by viral mediated expression of LCA. Unpaired two-tailed Student’s *t*-test demonstrated significant differences of expression of *TAC1*, *CALCB*, *TRPA1*, *TRPV1* and *SCN9A* genes between the LentiPirtLCA and LentiPirtGFP treated groups: *p* = 0.00003, 0.01173, 0.00236, 0.03726 and 0.03137, respectively. * *p* < 0.05; ** *p* < 0.01; *** *p* < 0.001. Samples in (**D**) are from three independent cultures (different samples from (**B**,**C**)).

Activated macrophages are known to play an important role in the pathogenesis and progression of rheumatoid arthritis, primarily through the secretion of various inflammatory mediators, such as TNF-α and IL-6 etc. These pro-algesic cytokines stimulate the expression and release of neuropeptide from sensory neurons [39,40], thus, contributing to the genesis of inflammatory and neuropathic pain. Herein, we thought to use the physiologically relevant concentrations of cytokines secreted from macrophage cells to treat sensory neurons to investigate the effect of expression of LCA in comparison to GFP on the transcription of pain-related genes. J774A.1 mouse monocyte macrophage cells were cultured for 24 h before being stimulated with 100 ng/mL of lipopolysaccharide (LPS) for 24 h. Cell supernatant containing secreted cytokines was collected. The concentration of secreted TNF-α and IL-6 were 5235.9 and 3989.9 pg/mL, respectively, by enzyme-linked immunosorbent assay (ELISA). Subsequently, mDRGs pre-treated with lentiPirtLCA or lentiPirtGFP at 5.4 × 10^4^ TU for 5 days were incubated with the LPS treated macrophage supernatant for 4 h before collecting the cells for RNA-seq (Figure 4A). In comparison to the lentiPirtGFP treated mDRGs, mRNA levels of *TAC1*, *HTR3A*, *GPR52*, *GM2808*, *NPY2R*, *ALK*, *IAPP* and *IGSF21* genes were significantly downregulated whereas mRNA levels of *CES1D*, *HFE2* and *COL9A2* genes were significantly upregulated (Figure 4B). Interestingly, viral-mediated LCA expression also lowered the transcriptional levels of *TRPV1*, *TRPA1*, *CALCB* and *SCN9A* genes, although the changes were less than 2-fold (Figure 4C). To verify the initial findings, we repeated the experiments. RT-qPCR was used to quantify the mRNA expression levels of these neuropeptide and transducer genes. Reassuringly, transcriptional levels of *TAC1*, *CALCB, TRPA1*, *TRPV1* and *SCN9A* genes were indeed downregulated by lentiPirtLCA with greater than 2-fold changes observed for substance P encoding gene (*TAC1*) (Figure 4D). The latter is in consistent with the RNA-seq results.

### 2.5. LentiPirtLCA Induced Persistent SNAP-25 Cleavage and Inhibition of K^+^-Evoked Substance P Release in the Infected Sensory Neurons

**Figure 5:**
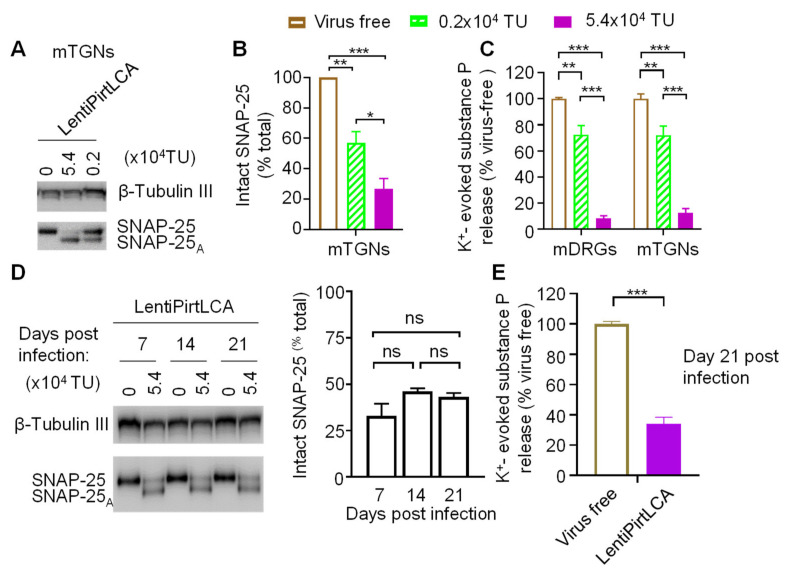
LentiPirtLCA yielded persistent cleavage of SNAP-25 and inhibition of K^+^-evoked substance P release from sensory neurons. (**A**) mTGNs were infected with lentiPirtLCA at the indicated doses for 5 days before probing the cleavage of SNAP-25. (**B**) The proportion of intact SNAP-25 remaining was calculated relative to the total (cleaved plus remaining intact). One-way ANOVA with Bonferroni’s multiple comparison test demonstrated significant differences between the groups: virus free vs. 0.2 × 10^4^ TU *p* = 0.00598; virus free vs. 5.4 × 10^4^ TU *p* = 0.00033; 0.2 × 10^4^ TU vs. 5.4 × 10^4^ TU *p* = 0.03006. (**C**) LentiPirtLCA inhibited K^+^-evoked substance P release from mDRGs and mTGNs. One-way ANOVA with Bonferroni’s multiple comparison test demonstrated significant differences between the groups in mDRGs: virus free vs. 0.2 × 10^4^ TU *p* = 0.00107; virus free vs. 5.4 × 10^4^ TU *p* < 0.00001; 0.2 × 10^4^ TU vs. 5.4 × 10^4^ TU *p* < 0.00001; in mTGNs: virus free vs. 0.2 × 10^4^ TU *p* = 0.00333; virus free vs. 5.4 × 10^4^ TU *p* < 0.00001; 0.2 × 10^4^ TU vs. 5.4 × 10^4^ TU *p* < 0.00001. (**D**) mDRGs were treated with lentiPirtLCA at 5.4 × 10^4^ TU/well. At day 7, 14 and 21 post viral infection, cells were harvested for probing SNAP-25 cleavage. Western blot demonstrated viral mediated LCA expression induced persistent cleavage of SNAP-25 (left panel). Right panel, quantified data derived from densitometric scanning of 4 independent cultures. One-way ANOVA with Bonferroni’s multiple comparison test demonstrated no significant differences between the groups (*p* > 0.05). (**E**) K^+^-evoked substance P release from LentiPirtLCA treated cells at day 21 after infection was normalized to the values obtained from virus-free control cells at day 21. Unpaired two-tailed Student’s *t*-test demonstrated LentiPirtLCA significantly inhibited substance P release (*p* < 0.000001), even at day 21 after infection. Note that, considering neuronal loss during cultivation, cell seeding density in (**D**,**E**) are ~3-fold of that in **C**. Data presented are from at least three independent cultures. * *p* < 0.05; ** *p* < 0.01; *** *p* < 0.001, ns, *p* > 0.05.

We have demonstrated that LentiPirtLCA can dose-dependently cleave SNAP-25 in infected mDRGs (Figure 2B,D). We repeated the same experiment with mTGNs. These were incubated with virus for 5 days before performing K^+^-evoked substance P release. Immunoblots and extent of cleavage quantified from densitometric scanning of three replicate gels reaffirmed the dose dependent cleavage of SNAP-25 in mTGNs by lentiPirtLCA (Figure 5A,B). To investigate the functional consequence of SNAP-25 cleavage by viral-mediated expression of LCA in mDRGs and mTGNs, K^+^-evoked substance P release was measured by ELISA. Cleavage of SNAP-25 by lentiPirtLCA resulted in dose-dependent inhibition of K^+^-evoked substance P secretion from the infected mDRGs and mTGNs (Figure 5C). Near ~90% inhibition of substance P release was obtained in mDRGs and TGNs infected by lentiPirtLCA at 5.4 × 10^4^ TU dose. Less inhibition of peptide release was observed at lower dose (Figure 5C).

The great advantage of using lentiviral vector is to yield long-term and stable expression of transgene. Herein, we used mDRGs as a cell-based model to monitor the cleavage of SNAP-25 over time by lentiPirtLCA. The ratio of intact SNAP-25 to LCA cleaved SNAP-25 virtually remained constant for 21 days (Figure 5D), the longest time we monitored. Most importantly, the K^+^-evoked substance P release at day 21 was still blocked by treatment with lentiPirtLCA virus (Figure 5E). Collectively, persistent cleavage SNAP-25 by vial-mediated LCA expression yielded long-lasting inhibition of K^+^-evoked substance P release from mDRGs.

## 3. Discussion

The present study successfully cloned a Pirt promoter which could selectively express exogenous genes in peripheral sensory neurons. When LCA gene was expressed in sensory neurons, SNAP-25 was cleaved and K^+^-evoked substance P release was inhibited. Importantly, viral-mediated LCA expression yielded persistent cleavage of SNAP-25 in sensory neurons and inhibition of substance P release, Thus, selective delivery of LCA gene into sensory neurons may provide a novel means for the treatment of chronic pain.

Gene therapy has advanced throughout the last two decades. Now gene therapy is also considered for many chronic diseases which significantly impact patients’ quality of life. Distribution of therapeutic gene is governed by the employed viral vector and the application sites. Joussain et al. used herpes simplex virus type 1 amplicon vectors to express BoNT LC gene by a non-specific promoter in cultured sensory neurons to inhibit CGRP release [41]. Although herpes simplex virus has tropism to peripheral sensory neurons, it has the risk to penetrate the neuromuscular junction, travel in a pure motor nerve to reach central nervous system motor nucleus [42]. Teng Q et al. demonstrated adenoviral vector mediated expression of tetanus toxin LC in the rat lumbar spinal cord disrupted hindlimb sensorimotor function [43]. In addition, in a rat model of seizures induced by motor cortex penicillin injection, adenoviral mediated LC expression in the cortex significantly reduced electroencephalogram frequency, amplitude, duration in epileptic rats and improved behavioral manifestations, without producing motor dysfunction when compared to control groups [44]. LC alone expressed by adenovirus cannot spread to other cells in the absence of the disulfide bridge bonded HC, but the broad transduction tropism of adenovirus to the majority of dividing cells and non-dividing cells (nerves) still poses the safety concern.

Pirt is expressed in most nociceptive neurons in DRG and TG (but not in the central nervous system) and is known to play an important part in sensing pain through modulation of TRPV1 channel [37]. Thus, Pirt gene promoter is suitable for expression of BoNT LC protease selectively in peripheral sensory neurons for management of chronic pain without producing motor dysfunction. Towards this, we have identified the Pirt promoter element and inserted into a lentiviral vector. Pirt promoter can effectively drive the expression of LCA in the DRG neurons but not in the spinal cord neurons. In addition, Pirt promoter has no activity in tested non-neuronal cells. It is noted that activity of cloned promoter is low compared to the conventional used human cytomegalovirus promoter, the latter is able to drive high level transgene expression in many mammalian cells. As high-level expression of a highly active proteolytic protease/enzyme is not needed, it may be advantageous to use low activity Pirt promoter because overexpressing an exogenous gene may result in negative effects on neuronal health. In this context, Pirt promoter activity is sufficient to express BoNT/A LC in sensory neurons infected by lentiPirtLCA as reflected by the rapid cleavage of neuronal target SNAP-25. The extent of SNAP-25 cleavage is related to the infection dose. Infection of cultured mouse DRGs and TGNs with lentiPirtLCA at ≥1.8 × 10^4^ TU (≥1.8 MOI), near all SNAP-25 was cleaved. At low MOI (<1 TU/cell), only a portion of SNAP-25 is cleaved. Interestingly, viral-mediated LCA expression in cytokine stimulated sensory neurons lowered the transcriptional levels of two major neuropeptide genes, *TAC1* and *CALCB* which encode substance P and CGRP, respectively, when compared to the cells treated with lentiPirtGFP. In addition, cleavage of SNAP-25 by the expressed LCA also downregulated mRNA levels of several transducer genes (e.g., *TRPV1*, *TRPA1* and *SCN9A*). Products of inflammation or nerve injury upregulate the expression and/or trigger sensitization of certain TRP and voltage-gated sodium channels, resulting in increased excitability (reduced threshold of activation) of nociceptors [45,46,47,48], thus, downregulation of their expression could result in increased threshold for detection of nociception.

Lentiviral-mediated Pirt promoter-driven expression of LCA yields persistent cleavage of SNAP-25. The ratio of cleaved SNAP-25 over intact in lentiPirtLCA infected sensory neurons remains unchanged for 21 days, the longest time point monitored in this study. This is striking different from the BoNT/A intoxicated sensory neurons. BoNT/A at 100 nM concentration cleaved more than 90% of SNAP-25 within 1 day. At day 6 post-intoxication, the extent of cleavage dropped to ~40% (Figure S1), revealing a relatively fast loss of cleaved SNAP-25 and recovery of full-length SNAP-25. This observation is unique because BoNT/A cleaved SNAP-25 lasts for months in other neuron types (e.g., spinal cord neurons, cerebella neurons etc.) [49,50]. The fast disappearing of BoNT/A cleaved SNAP-25 may be due to sensory neuron unique features. Neuropeptides (e.g., CGRP, substance P, etc.) are released from large dense-core vesicles at sites away from the active zones where exocytosis of classical synaptic vesicle occurs [51]. The more diffuse secretion enables cellular communication over wide areas. In addition, nerve growth factor may speed up the replenishment of membrane proteins and nerve regeneration. The rapid turnover of surface protein may quickly remove membrane-bound LCA [52], leading to the shortened duration of its action. Thus, the observed persistent cleavage of SNAP-25 in lentiPirtLCA infected sensory neurons apparently results from the viral-mediated constant expression of LCA leading to the long-lasting inhibition of substance P release.

In summary, for the first time, we successfully expressed the LCA gene in the sensory neurons using the identified Pirt gene promoter element. Decreasing the expression of pain neuropeptides and transducers as well as inhibiting neuropeptide release by viral-mediated LCA expression encourage future research on effects of this virus on relieving pain in animal models of chronic inflammatory and neuropathic pain.

## 4. Materials and Methods

### 4.1. Animals

C57BL/6 mice and Sprague-Dawley rats (originally sourced from SPF biotechnology co., LTD, Beijing, China) were bred in an approved Bioresource Unit at Henan University. Animals were housed 2 or 3 to a cage with ad libitum access to chow and water, under temperature-controlled conditions (21 °C–24 °C) on a 12 h light/dark cycle. Both sexes of postnatal day 2−3 C57BL/6 mouse pups and Sprague-Dawley rat were used in this study.

### 4.2. Design and Production of Lentiviral Vectors

A 928 bp synthetic fragment containing the Pirt gene promoter element was synthesized and subcloned into a previously reported dual promoter lentiviral vector [38] at NsiI and Nhe I sites to yield PirtGFP transfer vector. Subsequently, according to the published amino acid sequence of the clostridium botulinum type A-Hall strain (GenBank accession no.: AF488749.1, www.ncbi.nlm.nih.gov/nuccore/AF488749.1 accessed on 12 August 2021), LCA gene encoding residues 1-429 was synthesized and subcloned into the NheI and EcoRI sites to replace the GFP gene in PirtGFP transfer vector. The resulting transfer vector was named as PirtLCA. Production of recombinant lentiviruses (lentiPirtLCA and lentiPirtGFP) and determination of viral infectious titers were performed by the Hanbio Company (Shanghai, China) following standard procedures. Briefly, lentiviruses were successfully generated in 293T cells by co-transfection of the constructed transfer vector together with packaging (psPAX2) and envelope (pMD2.G) plasmids. Viruses in the harvested supernatant were concentrated by ultracentrifugation at 82,700 g for 120 min at 4 °C.

### 4.3. Culturing Macrophage Cells, Primary Human Keratinocytes (phKCs), Primary Neurons and Their Treatment with Lentivirus

RAW264.7 (Cat. No. CL-0190, Procell Life Science & Technology, Wuhan, China) and J774A.1 macrophage cells (Cat. No. CL-0370, Procell Life Science & Technology) and phKCs (Cat. No. 00192907, Lonza, Guangzhou, China) were cultured as previously described [53,54]. RAW264.7 and phKCs grown in 24 well plates were incubated with LentiPirtGFP for 5 days before being harvested for probing GFP expression.

TG, DRG and spinal cord are isolated from postnatal day 2−3 C57BL/6 mouse and Sprague-Dawley rat pups after being deeply-anesthetized with intraperitoneal injection of sodium pentobarbitone (60 mg/kg body weight) (Sinopharm Chemical Reagent Co., Ltd., Shanghai, China). Detailed procedures for dissociation and culturing of primary neurons are described before [55,56]. Isolated ganglia from each litter of pups were pooled together before dissociation. Briefly, ganglia were dissociated by dispase II (5 mg/mL) (Cat. No. 17105041, Thermo Fisher, Shanghai, China) and collagenase I (10 mg/mL) (Cat. No. 17018029, Thermo Fisher) at 37 °C for 30 min. The suspension was then gently triturated before adding 1 mg/mL DNase I (Cat. No. 10104159001, Merck, Shanghai, China) for 15 min. After centrifugation at 170× *g* for 5 min, the dissociated TG and DRG neuronal pellets were suspended, washed and cultured in medium (DMEM containing 5% (*v/v*) fetal bovine serum, 2% B27 (Cat. No. 17504044, Thermo Fisher), 100 IU/mL penicillin, 100 μg/mL streptomycin (Cat. No. 15140148, Thermo Fisher) and NGF (50 ng/mL) (Cat. No. 13257-019, Thermo Fisher)). Cells were seeded into 24-well plates precoated with poly-L-lysine and laminin (Cat. No. 11243217001, Merck) and maintained in a CO_2_ incubator at 37 °C. After 24 h and every other day thereafter, the culture supernatant was replaced with medium containing the anti-mitotic agent cytosine-β-D-arabinofuranoside (10 μM) (Cat. No. C1768, Merck). Spinal cord neurons isolation followed the published protocol [56]. Dissociated spinal cord neurons were cultured in neurobasal medium (Cat. No. 21103049, Thermo Fisher) supplemented with 2% B27, 0.5 mM GlutaMax, 100 IU/mL penicillin and 100 μg/mL streptomycin. Cultured primary neurons were incubated with or without lentiPirtLCA or lentiPirtGFP in the culture medium. After 24 h of culture, the cells were either immediately harvested in LDS-sample buffer or further cultured in the fresh medium for the indicated times before harvesting.

### 4.4. Quantification of K^+^-Evoked Substance P Release

Cultured primary sensory neurons pre-treated with or without lentivirus were incubated with low potassium basal release buffer (mM: 22.5 HEPES, 135 NaCl, 3.5 KCl, 1 MgCl_2_, 2.5 CaCl_2_, 3.3 glucose and 0.1% BSA, pH 7.4) at 37 °C for 30 min. Stimulated substance P release by high potassium buffer containing 60 mM KCl (isotonically balanced with NaCl) was performed in the same way. After brief centrifugation at 4 °C, the supernatants were stored at −80 °C. The amounts of substance P released were determined by enzyme immuno-assay following instructions for the kit (Cat. No. 583751, Cayman, Shanghai, China). K^+^-evoked release was calculated by subtracting the basal release from the high potassium buffer stimulated release.

### 4.5. Detection of Lentiviral Mediated GFP Expression and SNAP-25 Cleavage

LDS solubilized cell lysates were heated for 10 min at 100 °C and proteins were separated by SDS electrophoresis, using pre-cast NuPAGE 12% Bis-Tris gels (Cat. No. NP0342PK2, Thermo Fisher). Separated proteins were electrotransferred to polyvinylidene difluoride membranes. After blocking by 5% BSA in TBST buffer, membranes were incubated with primary antibodies for overnight at 4 °C. Mouse monoclonal antibodies against β-tublin III (1:2000, Cat. No. T8578, Merck) and β-actin (1:2000, Cat. No. A1978, Merck) were used to monitor protein loading for neuronal and non-neuronal lysates, respectively. Lentivirus mediated expression of GFP protein was probed with a rabbit GFP antibody (1:10000, Cat. No. ab183735, Abcam, Shanghai, China), whereas detection of SNAP-25 was performed using a mouse monoclonal antibody (clone SMI 81, 1:1000; Cat. No. 836304, Biolegend, Beijing, China) recognizing both intact and LCA cleaved SNAP-25. After extensive washing, specimens were incubated with horseradish peroxidase-conjugated anti-species secondary antibodies for 1 h. After washing, membranes were developed using chemiluminescence reagent (Cat. No. WBKLS0050, Merck), the lanes were analyzed using the gel documentation system (Syngene G: BOX Chemi XX9, Shanghai, China). For quantification of SNAP-25 cleavage, the proportion of full-length remaining was calculated relative to the total (cleaved plus remaining intact) using image J software analysis of captured images.

### 4.6. Immunofluorescence Staining

Murine dorsal root ganglionic neurons (mDRGs) grown in 24 well plates were infected with lentiPirtLCA (5.4 × 10^4^ TU/well, ~5.4 MOI) for 5 days before fixation with 3.7% formaldehyde. After washing, specimens were permeabilized in phosphate-buffered saline with 0.1% Triton X-100 and then incubated in PBS containing 1% BSA at room temperature for 1 h. Samples were then incubated with rabbit polyclonal antibody to TRPV1 (1:100, Cat. No. ACC-030, Alomone, Shanghai, China) together with mouse monoclonal antibody to the LCA cleaved SNAP-25 (1:500, Cat. No. MBS350064, MyBioSource, sourced from the representative company Biolead, Beijing, China) in blocking solution (4 °C, overnight). The specimens were washed in PBS and incubated with donkey anti-mouse Alexa 488 and donkey anti-rabbit Alexa 594. After the final wash of the secondary antibody, specimens were incubated with prolong anti-fade reagents containing 4′, 6-Diamidino-2-phenylindole dihydrochloride. Images were taken using a IX73 Olympus inverted microscope using CellSens Dimension Imaging software.

### 4.7. RNA-Seq and Reverse Transcription Quantitative Real-Time PCR (RT-qPCR)

Cultured J774A.1 macrophage cells in 24 well plates were stimulated with 100 ng/mL of lipopolysaccharide (LPS) (Cat. No. L4391, Merck) for 24 h at 37 °C. After brief centrifugation at 4 °C, the supernatants containing various cytokines were stored at −80 °C. The amounts of secreted TNF-α and IL-6 were determined by enzyme immuno-assay following instructions for the kits (Cat. No. DY410-05 and DY406-05, respectively, R&D Systems, Shanghai, China). Cultured mDRGs pre-treated with lentiPirtLCA or lentiPirtGFP at 5.4 × 10^4^ TU/well for 5 days were incubated with a mixture of DRG culture medium and the collected LPS-stimulated supernatant (500 μL each) at 37 °C for 4 h before collection for RNA-Seq. The latter was performed by BGI Group (Shenzhen, China). Briefly, total RNA was extracted from the cells using Trizol (Invitrogen, Carlsbad, CA, USA). Oligo (dT)-attached magnetic beads were used to purify mRNA. Purified mRNA was fragmented into small pieces with fragment buffer. Then, first-strand cDNA was generated using random hexamer-primed reverse transcription, followed by a second-strand cDNA synthesis. Afterwards, A-Tailing Mix and RNA Index Adapters were added by incubating to end repair. The cDNA fragments obtained from previous step were amplified by PCR, and products were purified by Ampure XP Beads, then dissolved in EB solution. The product was validated on the Agilent Technologies 2100 bioanalyzer for quality control. The double stranded PCR products from previous step were denatured and circularized by the splint oligo sequence to get the final library. The single strand circle DNA was formatted as the final library. The final library was amplified with phi29 to make a DNA nanoball (DNB) which had more than 300 copies of one molecule, DNBs were loaded into the patterned nanoarray and single end 50 bases reads were generated on a DNBseq platform (BGI-Shenzhen, China). Sequencing data are called raw reads or raw data, and quality control (QC) is then performed on the raw reads to determine whether the sequencing data are suitable for subsequent analysis. Sequencing data filtering was performed using software SOAPnuke developed by BGI independently (Parameters -l 15 -q 0.2 -n 0.05). After quality control, the filtered clean reads were aligned to the reference sequence. Hierarchical indexing for spliced alignment of transcripts is the software for mapping RNA-seq reads. After the alignment, the statistics of the mapping rate and the distribution of reads on the reference sequence are used to determine whether the alignment result passes the second QC of alignment. If it passes, we perform quantitative gene analysis and other analyses based on gene expression (principal component, correlation, differential gene screening, etc.). The resultant RNA-seq original data have been deposited to Gene Expression Omnibus (accession number: GSE176059). For RNA-seq, Log2 fold change was plotted and FDR (false discovery rate) was indicated. To verify the RNA-seq results using RT-qPCR method, extraction of total RNA from another three batches of the treated mDRGs was performed using TRIzol reagent (Cat. No. 15596026, Thermo Fisher). A total of 5 μg of RNA samples were reverse-transcribed into cDNA using the high capacity cDNA reverse transcription Kit (Cat. No. 4368814, Thermo Fisher) following the manufacturer’s instruction. qPCR was performed by incubating cDNAs with the SYBR Green PCR Master mix (Cat. No. 4309155, Thermo Fisher) with normalization to GAPDH levels. Primers for TAC1, CALCB, TRPA1, TRPV1 and SCN9A were bought from OriGene or synthesized by Sangon Biotech Co., Ltd. (Shanghai, China). PCR was performed using specific primers of mouse GAPDH, forward, 5-CATCACTGCCACCCAGAAGACTG-3, reverse, 5-ATGCCAGTGAGCTTCCCGTTCAG-3; TAC1, forward, 5-TAATGGGCAAGCGGGATGCTGA-3, reverse, 5-CCATTAGTCCAAC AAAGGAATCTG-3; CALCB, forward, 5-GAGGAGCAAGAGACTAAGGGCT-3, reverse, 5-GGCACAAAGTTGTCCTTCAGCAC-3; TRPA1, forward, 5-CCTTCCACAGAAGAC AAGTCCTG-3, reverse, 5-ACCACATCCTGGGTAGGTGCTA-3; TRPV1, forward, 5-CATCTTCACCACGGCTGCTTAC-3, reverse, 5-CAGACAGGATCTCTCCAGTGAC-3; SCN9A, forward, 5-TCCTTTATTCATAATCCCAGCCTCAC-3, reverse, 5-GATCGGT TCCGTCTCTCTTTGC -3.

### 4.8. Statistical Analysis

Statistical analysis and graphing were performed with Prism 7 software (GraphPad Software, San Diego, California, USA). All data are presented as means ± SEMs, *n* ≥ 3. When comparing more than two groups, One-way ANOVA with Bonferroni’s multiple comparison test was performed. When comparing two groups, statistical significance was assessed by unpaired two-tailed Student’s *t*-test. Probability values less than 0.05 was accepted as statistically significant. NS, non-significant with *p* > 0.05; * *p* < 0.05; ** *p* < 0.01; *** *p* < 0.001. For RNA-seq, Log_2_ fold change were plotted and FDR (false discovery rate) were indicated. FDR were determined and classified as being less than 0.001, ranging from 0.001 to less than 0.01, ranging from 0.01 to less than 0.05, or equaling 0.05 or more (non-significant).

## Data Availability

The RNA-seq original data have been deposited to Gene Expression Omnibus (accession number: GSE176059; reviewer token credentials: ynylassgdlghvax).

## Supplementary Materials

The following are available online at https://www.mdpi.com/article/10.3390/ijms22168826/s1.

## Abbreviations

BoNT	botulinum neurotoxins;
BoNT/A	botulinum neurotoxin serotype A;
CGRP	calcitonin gene-related peptide;
DRG	dorsal root ganglion;
ELISA	enzyme-linked immunosorbent assay;
FDR	false discovery rate;
LC	light-chain protease;
LCA	light-chain protease of botulinum neurotoxin A;
LPS	lipopolysaccharide;
mDRGs	murine dorsal root ganglionic neurons;
MOI	multiplicity of infection;
mTGNs	murine trigeminal ganglionic neurons;
Na_v_1.7	voltage-gated sodium channels 1.7;
phKCs	primary human keratinocytes;
Pirt	phosphoinositide-interacting regulator of TRP;
rDRGs	rat dorsal root ganglionic neurons;
rTGNs	rat trigeminal ganglionic neurons;
RFT-1	rabbit beta-galactoside alpha1,2-fucosyltransferase gene;
RT-qPCR	reverse transcription quantitative real-time PCR;
SCNs	spinal cord neurons;
SNAP-25	synaptosomal-associated protein 25-kDa;
SNAREs	soluble N-ethylmaleimide-sensitive factor attachment protein receptors;
TG	trigeminal ganglion;
TRP	transient receptor potential cation channels.
