# Supplementary material for: Selective Expression of a SNARE-Cleaving Protease in Peripheral Sensory Neurons Attenuates Pain-Related Gene Transcription and Neuropeptide Release
Source: Int J Mol Sci. 2021 Aug 17;22(16):8826. doi: 10.3390/ijms22168826 (PMC8396265; doi:10.3390/ijms22168826)
Supplement: Supplementary file 1 [file ijms-22-08826-s001.zip › ijms-1314061-supplementary.pdf]

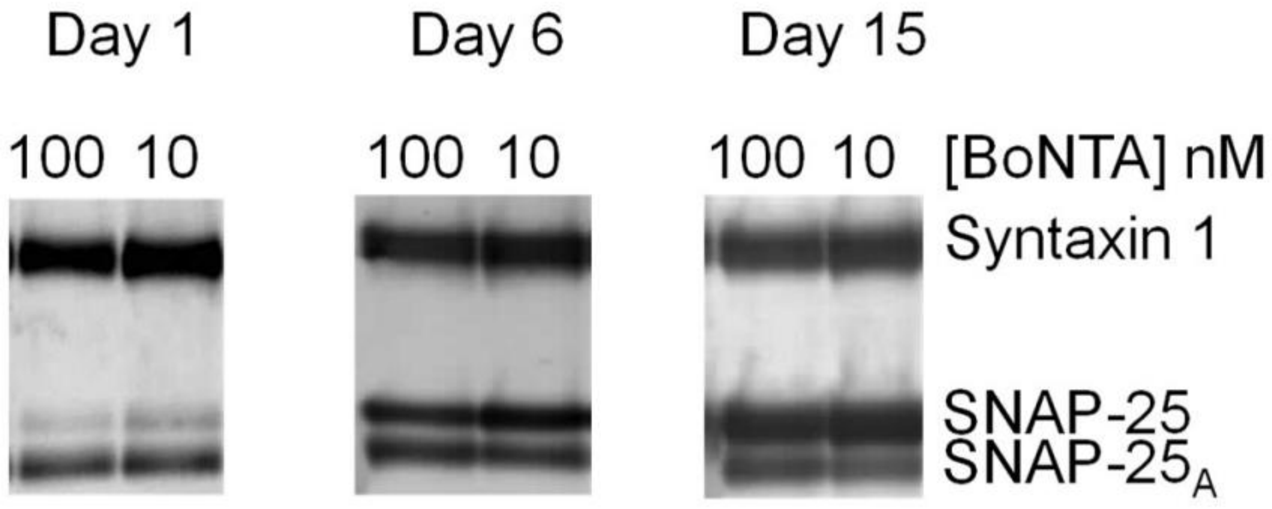

Figure S1. BoNTA-cleaved SNAP-25 decreases over time in the intoxicated trigeminal ganglionic neurons (TGNs). Cultured TGNs were intoxicated with BoNTA with the indicated concentrations for 24 h. Cells were either immediately harvested in LDS-sample buffer or continually cultured until the indicated time points before harvesting for probing the SNAP-25 cleavage. Syntaxin 1 was probed as an internal loading control. Representative blots revealing a relatively fast loss of cleaved SNAP-25 and recovery of full-length SNAP-25 in BoNTA intoxicated neurons.
